# Factors controlling nutrient availability to the developing fetus in ruminants

**DOI:** 10.1186/s40104-015-0012-5

**Published:** 2015-04-11

**Authors:** Kathrin A Dunlap, Jacob D Brown, Ashley B Keith, M Carey Satterfield

**Affiliations:** Department of Animal Science, Texas A&M University, 2471 TAMU, College Station, Texas 77843 USA

**Keywords:** Intrauterine growth restriction, Nutrient transport, Placenta, Ruminant

## Abstract

Inadequate delivery of nutrients results in intrauterine growth restriction (IUGR), which is a leading cause of neonatal morbidity and mortality in livestock. In ruminants, inadequate nutrition during pregnancy is often prevalent due to frequent utilization of exensive forage based grazing systems, making them highly susceptible to changes in nutrient quality and availability. Delivery of nutrients to the fetus is dependent on a number of critical factors including placental growth and development, utero-placental blood flow, nutrient availability, and placental metabolism and transport capacity. Previous findings from our laboratory and others, highlight essential roles for amino acids and their metabolites in supporting normal fetal growth and development, as well as the critical role for amino acid transporters in nutrient delivery to the fetus. The focus of this review will be on the role of maternal nutrition on placental form and function as a regulator of fetal development in ruminants.

## Introduction

It is widely accepted that maternal nutrient restriction during gestation results in offspring that are smaller at birth than counterparts from adequately fed mothers [1-4]. Sub-optimal fetal growth and development is a significant problem to livestock producers, contributing to lower productivity especially during periods of extreme nutritional hardship such as drought. The ability of the placenta to adapt to such environmental challenges influences nutrient transport to and development of the fetus. Understanding such differences will lead to elucidation of mechanisms for enhancing placental nutrient transport that will be necessary for generation of management strategies to combat fetal and neonatal loss.

In sheep, increasing the efficiency of livestock production can be more readily achieved by increasing the average number of offspring weaned than by improving growth rate or body composition [5]. Nearly one-half of all pre-weaning lamb deaths occur on the day of birth [6] with birth weight being the single greatest contributor to lamb mortality [7]. Even under moderate levels of management, death losses of more than 65% for lambs weighing less than 4 pounds at birth have been observed [8]. In comparison, lambs born weighing between 9 and 12 pounds exhibited losses of 6.4 to 8.1%. In cattle, analyses of over 83,000 births indicated that birth weight >1.5 standard deviations below the mean, within breed and sex, doubled the likelihood for perinatal mortality in both complicated and uncomplicated pregnancies [9]. The prenatal growth trajectory of all eutherians (placental mammals) is sensitive to the direct and indirect effects of maternal nutrition at all stages between oocyte maturation and birth [10-12].

### Fetal nutrient restriction

Pregnancy is a particularly sensitive period to environmental challenges that lead to suboptimal nutritional availability to the fetus due to the increased nutrient requirements for the dam and fetus. In cases of suboptimal nutrition the fetus may never be able to reach its maximal genetic potential [13]. Epidemiological studies have demonstrated links between maternal undernutrition/overnutrition and the susceptibility to chronic metabolic disease in adult offspring [14]. Metabolic syndrome has been defined as a cluster of disorders, including obesity, hyperglycemia [fasting serum glucose (*>*6.1 mmol/L)], hyperinsulinemia, hyperlipidemia, hypertension, and insulin resistance (impaired response of cells or tissues to physiological concentrations of insulin) [15]. These epidemiological observations have been recapitulated in controlled experimental studies using a variety of model systems, and have collectively given rise to the concept of fetal programming [16]. Fetal programming proposes that alterations in fetal nutrition and endocrine status impacts development, permanently changes structure and metabolism and, as a result, influences an individual’s susceptibility to disease [17]. Many of these changes are potentially mediated at the level of the epigenome, and are observed as stable and sometimes heritable alterations of genes through covalent modifications of DNA and core histones without changes in DNA sequences [18].

Maternal nutrition is arguably the most important and potentially manageable factor that contributes to pregnancy and maternal health [19]. Livestock species are produced in different environmental conditions. Swine are commonly produced in a confinement setting and are fed specifically formulated plant based diets (intensive systems). On the other hand ruminants, such as cattle and sheep, are more commonly managed under grazing systems. However, the quality of the grazing system is directly dependent on availability of nutrients, which is usually limited during the winter months and times of dry conditions. Thus nutrient availability may be limited for adequate growth and development as well as proper gestation and lactation [20]. The sheep, in particular, is a seasonal breeder that enters estrus in fall and early winter. Therefore, most of gestation occurs in the winter months, a time of low forage quality and limited nutrient availability [21]. In fact, Thomas and Knott reported, that without supplementation, the nutrient consumption of grazing ewes in the Western United States is often less than 50% of the National Research Council (NRC) recommendations (Council NR. Nutrient Requirements of Sheep. Washington, DC: Natl. Acad. Press; 1985.) In ruminants, IUGR remains a critical problem due to the traditional utilization of extensive forage based grazing systems which leaves the producer susceptible to large swings in nutrient quality and availability, coupled with challenges in feeding of supplemental nutrients in range based production systems [22]. Researchers and producers alike are working to find efficient and non-invasive means for preventing the continuous occurrence of IUGR and premature delivery. Thus nutritional means are an attractive option for non-invasive prevention. In order to fully utilize nutritional intervention one must understand the mechanisms by which nutrition mediates fetal growth.

Simplistically, it is easy to predict that more offspring lead to more total livestock product, and ultimately greater profit for the producer. Certainly, recent advances in the field of reproductive technologies, such as embryo transfer, have tested this theory. One would be quick to hypothesize that producing twins in cattle would be an easy way to increase profit; as the overhead costs for maintaining a single-calving cow accounts for more than 50% of the total costs of production [23]. Thus, one would assume that by doubling the amount of offspring produced by a single cow this would offer positive financial gains. However, this hypothesis does not recognize the impact of maternal reproductive health and adequate uterine space as well as management of a cow with two calves after birth. Cows that carry twins lose on average 12% body weight during the last trimester of gestation and twinning also reduces fetal growth and consequent birth weight of offspring. This could lead to decrease weight at sale. Thus, careful consideration must be demonstrated in analyzing enhanced production technologies. The sheep industry displays a similar situational paradigm. Under ideal environmental and management conditions multiple offspring pregnancies increase the prolificacy of the species. However, it is important to note, that increased number of fetuses can contribute to placental insufficiencies and thus lower birth weights [24]. As mentioned, prolificacy is an important factor in intensive sheep production, however the economic benefits of high prolificacy ewes are usually not fully exploited because multi-fetal pregnancies are associated with IUGR, increasing perinatal mortality and altering postnatal growth trajectories.

Other natural factors can affect birth weight in sheep, such as maternal age. Lambs born to relatively young ewes and relatively old ewes generally possess lower birth weights [25]. In young animals the fetus is in direct competition for nutrients with the growing dam as opposed to older ewe, which typically have reduced body condition scores, and thus may struggle to balance the nutritional needs for survival as opposed to growth. Female body condition at mating may also influence fetal development, as ewes that were lighter and thinner at mating, and thus exhibited low nutrient intake, had lambs with an associated 13% decreased birth weight [26]. Seasonality can also be an indicator of birth weight. Lambs born in the fall and summer are generally lighter than those born in the spring and winter; which can possibly be explained by alterations in melatonin and seasonal effects on gestation. Additionally, gestation occurring in the warmer months where ewes shuttle blood flow to the periphery of the body to dissipate heat rather than to the core could ultimately decrease placental blood flow and placental growth [27].

Altitude is another factor that affects birth weights. Offspring that are born at high altitude may experience hypobaric hypoxia and be lighter than those born at a lower altitude. Heat stress is a primary concern in cattle production, particularly relevant to the dairy industry. Heat stress has been shown to reduce placental weight and mass [28-30] while also negatively impacting the hormonal properties of the placenta, having decreased levels of placental lactogen and pregnancy associated glycoprotein [29,31].

All of these situations can ultimately lead to IUGR offspring who possess reduced survival ability [25]. Ultimately, the hypothesis is that exposure to any of these challenges may cause alterations in fetal growth stemming from a hindrance of the placental growth trajectory.

### Placental formation

The placenta mediates the transport of nutrients, gases, and waste products of their metabolism between the maternal and fetal circulation [22]. Placental growth precedes fetal growth and its development is crucial for optimal fetal growth [32-34]. The placenta has two evolutionary roles conserved among all species. The first is it generates a large surface area for nutrient exchange by the epithelial barrier and fetal blood vessels, all of which make up the chorioallantoic placenta. The second basic function is that the trophoblast cells interact closely with the uterus, which produces histotroph that provides key growth factors, nutrients, and immune cell regulators necessary to facilitate blood flow and nutrient delivery to the fetus [35]. In addition to the presence of secretory uterine glands, the ruminant uterus is also home to a number of aglandular areas of stroma that are covered by a single layer of luminal epithelial cells, which are termed caruncles. The number of caruncles varies between species, with sheep having approximately 100 and deer less than 10 and cattle ranging between 75–125 [32,36]. During pregnancy the carunclar crypts will interdigitate with the fetal cotyledonary villi to give rise to a feto-maternal structure known as a placentome, which is the structure responsible for high-throughput nutrient transfer between the uterus and the fetus [37]. Failure of placentome development results in loss of the fetus [38], because they provide the primary source of hematotrophic nutrition as maternal and fetal blood vessels are in very close proximity for exchanging oxygen and micronutrients [39]. Vascularization of the placentomes is established early in gestation, which is to be expected as the majority of placental growth occurs during the first half of pregnancy [34]. Increased microvascular density, interdigitation and alterations in villous shape are potential mechanisms by which the placentome adapts to the maternal environment to meet the growing demands of the fetus [40]. As these structures are responsible for an increasing percentage of blood flow throughout gestation it is not surprising that they continue to experience modest changes in capillary area density from mid to late gestation [41,42]. As stated, failure of placentome formation results in loss of pregnancy, however a surgical reduction in the number of caruncles results in an increase in the average size of placentomes [43]. This may prove to be a compensatory mechanism by which the uterus and placentomes work to provide support required for fetal development. In the bovine uterus a relatively small number (20) of cotyledons are first visible on the fetal placental membrane as early as day 37 of pregnancy, however that number has tripled by day 50 and the beginning of a caruncular- cotyledonary interrelationship is clearly present [44,45]. By day 90 of pregnancy there are greater than 100 placentomes present in the bovine uterus, each possessing a characteristic mushroom-like shape, rooted with a stalk like structure stemming from the original caruncle [46]. Similarly, by gestational day 40 in the sheep and the goat, there is maximal juxtaposition of endometrial and placental microvasculatures [35,47]. As pregnancy progresses and the placentome continues to develop, the principal layers of the fetal capillary, trophoblast epithelium, crypt lining, and maternal capillary remain distinctive [47]. The general morphological characteristics of the placentomes are maintained as pregnancy progresses with the degree of interdigitation of the tissues becoming greater.

A great deal of research has been conducted in the ewe with respect to the interaction with nutrition and placental development and morphology. Recognizing that placental development is a major indicator of birth weight, researchers have found an ever occurring commonality among studies: the morphology of the placenta is altered under nutrient scarce conditions, with the most notable change being increased development of the fetal cotyledon. This change is thought to reflect an adaptive compensatory response to maximize transplacental exchange and thus fetal growth [48-50]. In ovine models of nutritionally induced fetal growth restriction there is an inverse relationship between nutritional plane and fetal vascular development [3,51,52]. These morphological changes are expected to diminish fetal development through altered availability of hematotrophic support.

### Placental blood flow

During pregnancy, transport of nutrients from the mother to the fetus is predominantly dependent on uterine blood flow. Indeed, fetal and placental weights and uterine blood flow are highly correlative. In addition, factors that alter fetal weight such as genotype, maternal nutrient intake, environmental heat stress, high altitude, and fecundity, result in parallel alterations in placental weight and uterine blood flow [53]. In the sheep, uterine and placental blood flows progress in a non-linear pattern with an early rise in blood flow between days 40–70 as the placental cotyledon develops and a second increase in flow between days 120–140 [54]. This early rise in blood flow is likely critical in the development of a functional, efficient placenta. Importantly, 85% of blood flow to the gravid uterus is directed to the placentome [55]. A critical regulator for vascular permeability, blood flow, and capillary growth is the angiogenic factor, vascular endothelial growth factor (VEGF). In the developing bovine placentome, VEGF and its receptors FLT1 and KDR are abundantly expressed [56]. Specifically VEGF is present in the trophoblast giant cells, fetal endothelia and maternal endothelia, which also expresses high amounts of FLT and KDR antigen. The sheep exhibits a similar pattern of expression with placental VEGF mRNA present in both the fetal and maternal endothelia as well as the chorionic epithelium [57]. Placental expression of VEGF and the receptors in a model of placental insufficiency-intrauterine growth retardation (PI-IUGR) indicates that placental growth, as indicated by weight, ceases at day 90 of pregnancy in the sheep and declines to term [58]. However, placental transport capacity continues to increase to meet the requirements of the exponentially growing fetus as evidenced by the late rise in uterine blood flow during the latter stages of gestation [59]. Importantly, the ability to respond to the increased demand for nutrients during late gestation can be undermined by poor placental development earlier in gestation. In the sheep, maternal undernutrition results in a 14% decrease in caruncular capillary area density in late pregnancy [53]. Mid-gestational nutrient restriction in the sheep results in a decreased abundance of mRNA encoding VEGF in placentomes [60]. These morphological and vascular changes reduce hematotrophic support and diminish fetal development. Changes in microvascular density and vessel diameter are also commonly found in compromised pregnancy [51,61,62].

### Placental fluids

The ruminant placenta is comprised of two distinct fluid-filled membranes: the amnion and allantois, which act as reservoirs for nutrients that are essential for fetal growth and development [63-65]. Maternal nutrient restriction during mid pregnancy in sheep results in decreased allantoic and amniotic fluid volume [60]. In sheep, fetal growth retardation, induced by maternal nutrient restriction is associated with decreased quantities of serine, α-amino acids, arginine family amino acids, and branched chain amino acids in both amniotic and allantoic fluid [1,2]. Arginine and its precursor, citrulline, are abundant in ovine uterine fluids [66]. Swallowing of amniotic fluid provides a rich source of nutrients for utilization by the fetal intestine and other tissues [67]. Prevention of amniotic fluid entry into the small intestine by esophageal ligation results in fetal IUGR in sheep [63]. Allantoic fluid is primarily derived from placental transport mechanisms [68] and in sheep, the volume increases in early gestation (days 25–40) before decreasing briefly (days 40–70) prior to increasing steadily until near term (day 140) [32]. It is now clear that allantoic fluid nutrients may be absorbed by the allantoic epithelium into the fetal-placental circulation and utilized by fetal-placental tissues [68]. The allantoic fluid during early to mid-gestation in the sheep is an abundant source of arginine and polyamines [69]. Allantoic fluid is also a reservoir for both glucose and fructose in the sheep, and both concentrations and total levels of fructose are much greater than that of glucose and change significantly with day of gestation [32].

### Placental nutrient transport

Placental nutrient transport is required for fetal growth [70,71]. Realizing that most fetal growth occurs during the final third of pregnancy, the development of the placental networks regulating transfer must precede that time point [40]. Although placental blood flow is imperative for optimal nutrient delivery, expression and/or activity of specific transporters is the rate limiting step for delivery of many nutrients, including glucose and amino acids [72]. Numerous environmental factors, such as, under- and over- nutrition, hypoxia, heat stress, and hormone exposure may regulate activity of both glucose and amino acid transporters in the placenta [73-79]. In other species, compromised pregnancies are associated with specific alterations in transporter availability and function [77,80]. In rats maternal dietary protein deprivation results in down regulation of placental amino acid transport systems prior to the emergence of fetal growth restriction [81]. In sheep, maternal infusion of mixed amino acids results in variable changes in umbilical uptake depending on the amino acid [71] highlighting a complex system of amino acid transport likely involving transporter availability, capacity, promiscuity, affinity, and competition. Sheep models of placental insufficiency induced by heat stress, indicate that umbilical uptake of essential amino acids is reduced, however fetal amino acid concentrations are unaltered compared to controls [82]. To date, the literature regarding the effect of maternal nutrient restriction on placental transporter availability and function is limited.

### Select nutrients and fetal growth

Fetal growth and metabolism is dependent upon glucose as it is the primary energy substrate for the placenta and fetus and supplied by the maternal bloodstream [83]. As the fetus grows, so does its rate of absolute glucose utilization and thereby the placental rate of glucose transfer must increase accordingly. Changes in maternal glucose levels and/or rate of placental transfer require the fetus to adapt metabolically to maintain consistent levels for its trajectory of development [83]. Fructose, while not as intensely investigated in the ruminant placenta, also appears to play a role in pregnancy in the sheep, perhaps as a mediator of the mammalian target of rapamycin (MTOR) cell signaling pathway [32,84,85].

It is established that amino acids play a vital role in development of the conceptus (embryo/fetus and associated placental membranes). In addition to serving as building blocks for tissue protein synthesis, amino acids function as antioxidants, regulators of hormone secretion, major fuels for fetal growth, and cell signaling molecules [86,87]. Amino acids are also essential precursors for the synthesis of non-protein substances with biological importance, including nitric oxide, polyamines, neurotransmitters, amino sugars, purine and pyrimidine nucleotides, creatine, carnitine, porphyrins, melatonin, melanin, and sphingolipids [88,89]. Nitric oxide (NO), a product of arginine catabolism, plays a crucial role in regulating placental angiogenesis and fetal-placental blood flow during gestation [90-92]. Polyamines (polycationic molecules synthesized from ornithine) regulate gene expression, signal transduction, ion channel function, and DNA and protein synthesis, as well as cell proliferation, differentiation, and function [87]. It should be noted that a comprehensive series of studies investigating the role of select nutrients and their transporters in the developing ruminant conceptus during the early peri-implantation period have been published [66,93-98], however discussion of these studies is beyond the scope of this review.

Studies from our laboratories have previously shown that maternal nutrient restriction results in the reduction of amino acids and polyamines in fetal umbilical venous plasma as well as amniotic and allantoic fluid [1,2]. Interestingly, inhibition of ornithine decarboxylase (ODC), which converts ornithine to putrescine (a polyamine), during mid-pregnancy in mice results in embryonic growth arrest and impaired development of the yolk sac and placentae [99], while gene ablation results in embryonic lethality prior to gastrulation [100]. In rats, inhibition of ODC activity results in fetal IUGR as well as a reduction in placental weight and placental DNA content [101]. Interestingly, despite a wealth of information highlighting the importance of polyamines in physiology the transport mechanisms of these molecules have not been elucidated in mammals. As a first step in ameliorating IUGR using intervention strategies we administrated sildenafil citrate (Viagra) to pregnant sheep, which resulted in increased fetal growth in both nutrient-restricted and well fed controls [2]. This increase in fetal growth was coordinate with increased concentrations of amino acids and polyamines in the amniotic and allantoic fluids as well as the fetal circulation [2]. As sildenafil citrate is not a viable strategy for production agriculture we performed subsequent studies with nutritional intervention. Intravenous administration of arginine similarly prevented IUGR in underfed ewes and increased percentage of lambs born alive in ewes carrying multiple fetuses [102-104]. These data indicate that amino acids and polyamines are essential for enhanced fetal growth during maternal undernutrition. It is hypothesized that arginine could be increasing both NO for vasodilation as well as providing a substrate for polyamine production. Further it may play an essential role in activation of the MTOR cell signaling pathways, stimulation of placental growth and mediation of placental blood flow [105].

### Localization of glucose transporters within the placentome

A multitude of membrane transporters, primarily those belonging to the solute carrier (SLC) superfamily, may be found on the placenta to facilitate nutrient transport to the fetus. Classification of these transporters is based on their structure and substrate preference [97,106,107]. Transporter localization at both the maternal and fetal surfaces for transport across plasma membranes is essential in the delivery of glucose, amino acids, polyamines, and other nutrients from maternal circulation to umbilical circulation [108]. Work in various species, such as sheep, humans, and rodents, has illustrated the necessity for placental nutrient transporters throughout gestation [66,71,77,81,95-97,106,108,109].

A comprehensive microscopy study of ruminant placentomes (including: sheep, goats, cattle and deer) revealed the specific localization of two primary glucose transporter isoforms, SLC2A1 and SLC2A3 [110]. SLC2A1 is expressed on the inner and outermost membranes of the placenta between the fetal and maternal endothelia while SLC2A3 is expressed only by trophoblast microvilli, suggesting that in order for successful glucose transport between the mother and the fetus both SLC2A1 and SLC2A3 must be utilized sequentially [110]. While localization remains consistent, there is a temporal shift in the abundance of expression of *SLC2A1* and *SLC2A3* in the sheep placenta, with *SLC2A3* levels increasing late in gestation [111]. The change in relative levels of glucose transporter expression suggests that SLC2A3 may play a critical role in mediation of glucose transport late in ruminant gestation. Interestingly mid-gestational maternal nutrient restriction does not influence relative abundance of *SLC2A1* nor *SLC2A3* mRNA in the sheep placenta [60]. Lactation also places a strain on the maternal metabolic system. In the case of lactating dairy cows, blood glucose levels are lower in comparison to their non-lactating contemporaries [112]. Regardless of lactational status, expression of *SLC2A1* and *SLC2A3* was most abundant in the placenta as opposed to liver or endometrial tissues in early gestation, with relative levels of *SLC2A3* in the placenta progressing with pregnancy [112].

### Expression of amino acid transporters within the placentome

**Figure 1:**
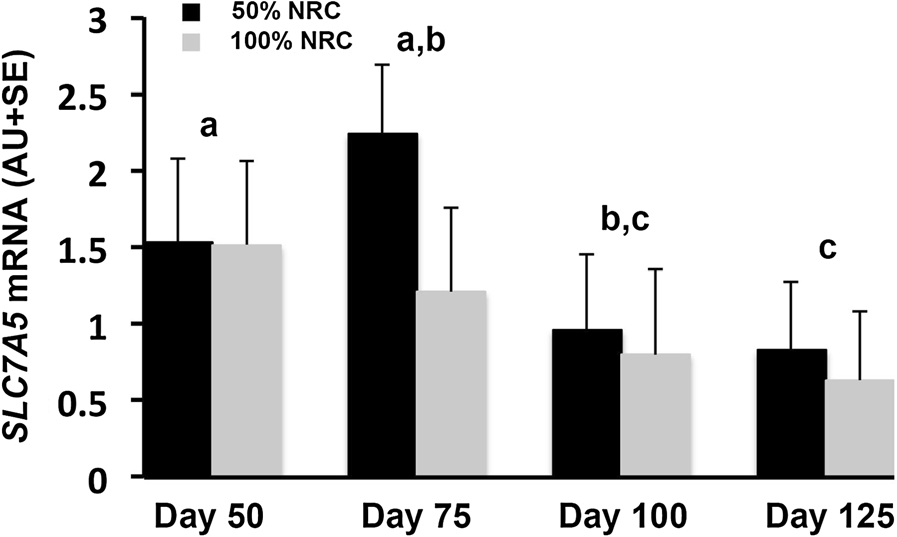
Steady-state mRNA levels of the large neutral amino acid transporter S*LC7A5*. Results indicate that mRNA expression of *SLC7A* is higher (*P* < 0.05) in placentomes from ewes on day 50 of pregnancy as compared to day 100 and 125 and that levels are similarly greater on day 75 than 125. Columns lacking a similar letter differ statistically (*P* < 0.05).

**Figure 2:**
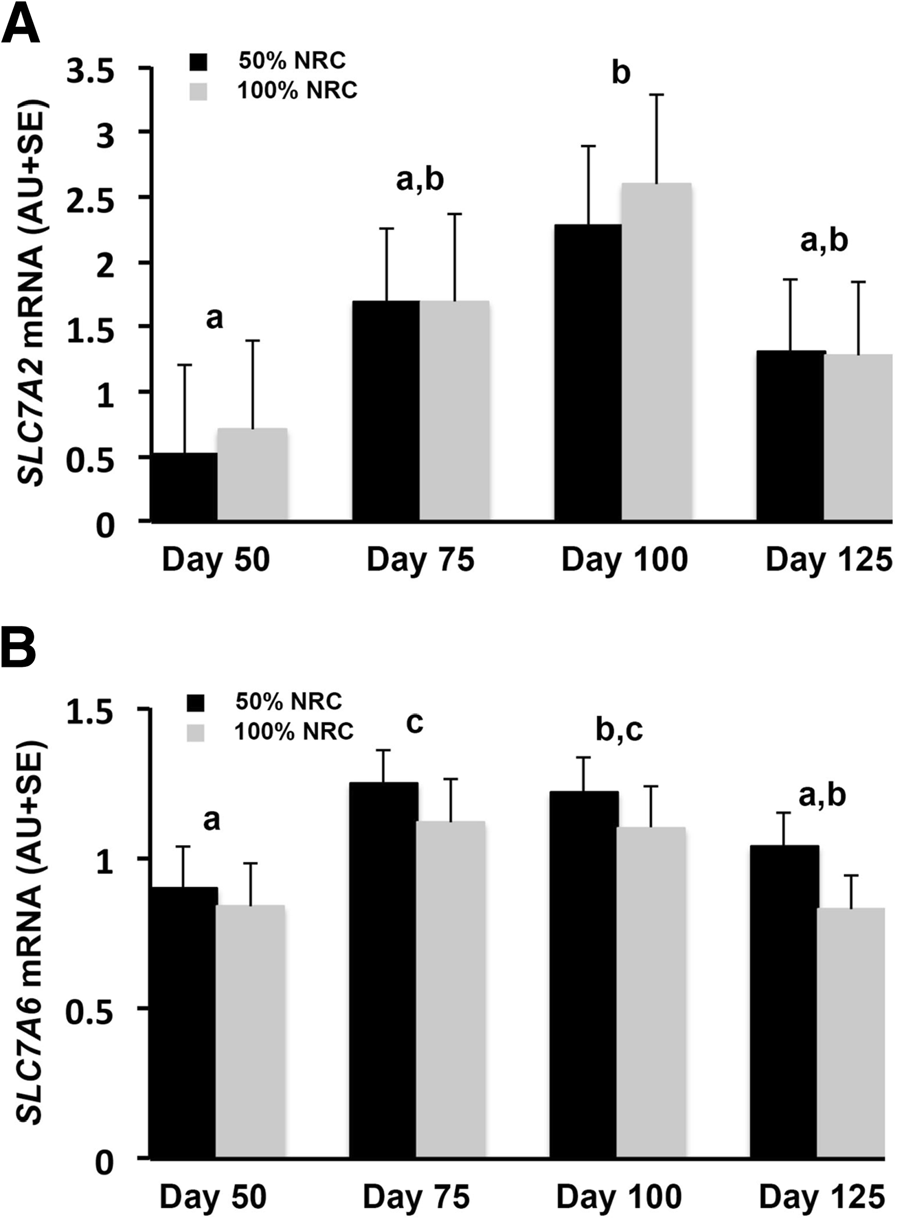
Steady-state mRNA levels of the cationic amino acid transporters (**A**) *SLC7A2* and (**B**) *SLC7A6* are presented. Results indicate that mRNA expression of *SLC7A2* and *SLC7A6* are higher (P < 0.05) in placentomes from day 100 than day 50. Results also indicate that *SLC7A6* mRNA levels are lower in placentomes from day 125 than 75. Columns lacking a similar letter differ statistically (*P* < 0.05).

**Figure 3:**
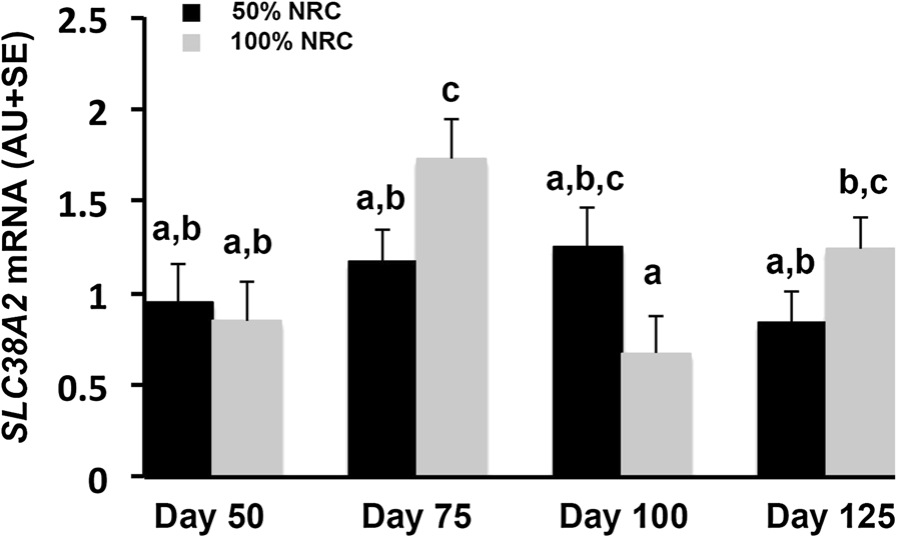
Steady-state mRNA levels of the sodium coupled neutral amino acid transporter (SNATs) *SL38A2* is presented. Results indicate that mRNA expression of *SLC38A2* is not different from days 50 to 125 in nutrient restricted ewes (*P* > 0.1), however in placentomes of well-fed ewes SLC38A2 increases from days 50 to 75, decreases to day 100 and then increases to day 125 (*P* < 0.05).

Although placental blood flow is imperative for optimal nutrient delivery, expression and/or activity of specific transporters is the rate limiting step for delivery of many nutrients, including glucose and amino acids [70,72]. In both humans and rats, compromised pregnancies are associated with specific alterations in transporter availability and function [77,80]. In rats, maternal dietary protein deprivation results in down regulation of placental amino acid transport systems prior to the emergence of fetal growth restriction [77,81]. The sheep has been a well-utilized model for investigation of amino acid transporter profile in early gestation [66,95,96] as well as manipulated models of pregnancy [113,114]. The observations in the literature support results from our laboratory investigating changes in placentome amino acid transporter expression over the duration of pregnancy as well as in response to maternal nutrient restriction. Using an experimental model developed in our laboratory [102] and methods for quantification of steady-state mRNA levels previously published [102] we investigated expression of the amino acid transporters shown in Table 1. Our results show that stage of pregnancy impacts relative expression of multiple amino acid transporters. Specifically, steady-state mRNA levels of the large neutral amino acid transporter, *SLC7A5*, (Figure 1) demonstrate that expression is impacted by day of pregnancy rather than maternal nutrient status. Levels of *SLC7A5* are higher (*P* < 0.05) at day 50 than at days 100 and 125, with the most abundant level in the nutrient restricted ewes occurring at day 75. Steady-state mRNA levels of cationic amino acid transporters are shown in Figure 2. *SLC7A2* mRNA levels increased (*P* < 0.05) in placentomes from days 50 to 100 and were not impacted by maternal nutrient restriction. *SLC7A6* mRNA levels were higher (*P* < 0.05) in placentomes from days 75 and 100 of pregnancy as compared to days 50 and 125 (Figure 2). *SLC7A7*, *SLC7A8,* and *SLC7A1* mRNA levels in placentomes did not differ (*P* > 0.10) over the course of gestation or in response to maternal nutrient restriction. Unlike the previously described amino acid transporters a sodium coupled neutral amino acid transporter (SNAT) SLC38A2 exhibited a day by diet interaction (Figure 3). Specifically, *SLC38A2* mRNA levels in placentomes of well- fed ewes increased from days 50 to 75, decreased to day 100 and then increased again to day 100 (*P* < 0.05). In nutrient restricted ewes there was no difference thoughout the course of gestation. In contrast, *SLC38A4* mRNA levels in placentomes were not different (*P* > 0.10) between groups.

**Table 1:** **Primers utilized for quantitative real-time PCR analysis**

**Target** ^**1**^	**Forward/revers primers, (5’→3’)** ^**2**^	**Length of amplicon, bp**	**GenBank accession No** ^**3**^
SLC7A1	CCTAGCGCTCCTGGTCATCA	56	AF212146
	GGGCGTCCTTGCCAAGTA		
SLC7A2	GCAGAGCAGCGCTGTCTTT	62	XM_002698665
	ACTGTCCAGAGTGACGATTTTCC		
SLC7A5	GGTGAACCCTGGTACGAATTTAGT	64	NM_174613
	TCCACGCTCGAGAGGTATCTG		
SLC7A6	CATTTGTGAACTGCGCCTATGT	72	NM_001075937
	CCAGGACCTTGGCATAAGTGA		
SLC7A7	TCAGGCTTGCCCTTCTACTTCT	64	NM_001075151
	GGAGCCAAAGAGGTCGTTTG		
SLC7A8	GGCCATGATCCACGTGAAG	65	NM_001192889
	GGGTGGAGATGCATGTGAAGA		
SLC38A2	CAGCTATAGTTCCAACAGCGACTTC	77	NM_001082424
	CATCGGCATAATGGCTTTTCA		
SLC38A4	TGCTTCATGCTTACAGCAAAGTG	63	NM_001205943
	CAGCCAGGCGTACCATGAG		

^1^The amplification target.

^2^The forward and reverse DNA oligos used in the amplification of the target. Forward and reverse primers do not necessarily indicate the *in vivo* direction of transcription.

^3^The accession number to the ovine or bovine sequence that was used during primer design.

These data suggest that while there is a marked temporal change in expression the impact of maternal nutrition on transporter expression is more subtle. We have observed in previous studies that the existence of a subpopulation of animals that support normal rates of fetal growth despite maternal nutrient restriction. This would suggest the ability of the placenta to adapt, in certain cases, to maternal nutrient restriction. Specifically that there is a sub-population of animals that even in cases of maternal undernutrition undergoes an adaptive placental response that supports development of fetal weight at a level similar to well fed controls. Indeed, based on prior observations, our laboratory conducted a series of studies to identify a population of IUGR and non-IUGR offspring from a similar cohort of nutrient-restricted ewes as a first step to assess adaptive mechanisms of placental nutrient transport. Briefly, we have observed that the range of fetal weights is greater from ewes receiving only 50% of their NRC requirements than for ewes fed to meet 100% of their nutrient requirements, when confounding variables such as maternal size, genotype, and fecundity are controlled (Satterfield and Dunlap, unpublished results). Suggesting that there is a mechanism by which the placenta adapts to maternal nutrient restriction to support normal versus restricted fetal growth in an outbred population as opposed to laboratory animals with less heterogeneity. These results are similar to those observed in beef cattle whereby maternal nutrient restriction from early to mid gestation resulted in two distinct groups of IUGR and non-IUGR fetuses at mid gestation [115]. Those IUGR pregnancies were also characterized by smaller cotyledonary weights and reduced placentomal surface area [115] which is similar to results of our studies, and further supporting a large body of literature indicating that placental weight is positively correlated with fetal weight. Further supporting the impact of population variation in maternal response to undernutrition are studies conducted using the heterogenous population of Western white-faced ewes adapted to harsh climates. In these ewes fetal growth to day 78 was not affected by maternal nutrient restriction [49]. Amino acid and polyamine concentrations in the fetal circulation of these sheep are maintained despite maternal nutrient restriction [116]. Assessment of amino acid transporter expression comparing the IUGR to non-IUGR pregnancies identified a number of amino acid transporters that were differentially expressed between the two groups, including *SLC7A6, SLC7A7, SLC7A8, SLC38A2*. Expression of those transporters were greater in IUGR than non-IUGR pregnancies (Satterfield and Dunlap, unpublished observations). These data indicate that while amino acids and polyamines are necessary for fetal growth the mechanisms regulating their transport and utilization may vary greatly amongst populations.

## Conclusions

Optimal fetal growth requires the efficient delivery of nutrients to the fetus and corresponding removal of waste products associated with fetal metabolism and growth. The mechanisms by which fetal nutrient delivery is achieved are well accepted and include hematotrophic nutrition, histotrophic nutrition (secretions emanating from the uterine glands), placental metabolism of substrates for use by the fetus, and the activity of nutrient transport systems within the placenta. These mechanisms work in concert to provide sufficient quantities of nutrients to the fetus for growth. Perturbations in any of these mechanisms have significant impacts on the growth and well-being of the fetus. Collectively, results of the presented studies coupled with data from the existing literature suggests that the placenta is a dynamic organ, whose form and function can be regulated by a myriad of factors. Further, results support previous findings from our laboratory and others, and highlight roles for amino acids and their metabolites in supporting normal fetal growth and development, as well as the critical role for amino acid transporters in nutrient delivery to the fetus. Further research is needed to address a series of mechanistic questions in order to increase understanding of appropriate nutrient delivery to the fetus. In the case of the adaptive ruminant placenta, is the difference in fetal growth a response of increased uterine blood flow, an alteration in early placental development, an adaptive recruitment of additional nutrient transporters or an increase in their activity, or some combination of these factors? It is important to utilize such models for future investigation of placental adaptation employed in an effort to increase nutrient delivery to the fetus despite limited maternal nutritional intake.

## Abbreviations

IUGR: Intrauterine growth restriction
NRC: National Research Council
VEGF: Vascular endothelial growth factor
FLT: Vascular endothelial growth factor receptor −1
KDR: Vascular endothelial growth factor receptor −2
PI-IUGR: Placental insufficiency intrauterine growth restriction
NO: Nitric oxide
ODC: Ornithine decarboxylase
MTOR: Mammalian target of rapamycin
SLC2A1: Solute carrier family 2 member 1
SLC2A3: Solute carrier family 2 member 3
SLC7A2: Solute carrier family 7 member 2
SLC7A5: solute carrier family 7 member 5
SLC7A6: Solute carrier family 7 member 6
SLC7A7: Solute carrier family 7 member 7
SLC7A8: Solute carrier family 7 member 8
SNAT: Sodium coupled neutral amino acid transporter
SLC38A1: Solute carrier family 38 member 4
SLC38A4: Solute carrier family 38 member 4
